# Postnatal maternal mental health-related hospitalisation and its association with adverse child health and maltreatment outcomes: narrative review

**DOI:** 10.3389/fpsyt.2026.1850017

**Published:** 2026-06-08

**Authors:** Demeke Mesfin Belay, Abel Fekadu Dadi, Yohannes Tesfahun Kassie, Wubet Alebachew Bayih, Behailu Derseh, Binyam Minuye Birhane, Bernard Leckning, Steven Guthridge

**Affiliations:** 1 Menzies School of Health Research, Charles Darwin University, Darwin, NT, Australia; 2 College of Health Science, Debre Tabor University, Debre Tabor, Ethiopia; 3 Addis Continental Institute of Public Health, Addis Ababa, Ethiopia; 4 Department of Epidemiology and Preventive Medicine, School of Public Health and Preventive Medicine, Faculty of Medicine, Nursing and Health Science, Monash University, Melbourne, VIC, Australia; 5 Health Science College, Debre Birhan University, Debre Birhan, Ethiopia; 6 School of Public Health, University of Technology Sydney, Sydney, NSW, Australia; 7 Discipline of Psychiatry and Mental Health, School of Clinical Medicine, Faculty of Medicine and Health, University of New South Wales, Sydney, NSW, Australia

**Keywords:** adverse health, child, hospitalisation, maltreatment, maternal mental health, postnatal

## Abstract

**Background:**

Maternal mental health conditions within the postnatal period can have long-lasting consequences on a child’s well-being. This study synthesises current evidence on the association between postnatal maternal mental health-related hospitalisation (MHrH) and adverse child health and maltreatment outcomes.

**Methods:**

We conducted an inclusive search across multiple databases, including MEDLINE, PsycINFO, CINAHL, Scopus, Embase, Google Scholar, and the reference list of eligible papers. Studies that used standardised outcome measures were included. Study selection and data extraction were made using a standardised Joanna Briggs Institute (JBI) tool. The quality of the studies was assessed using the Newcastle-Ottawa Scale (NOS). Descriptive vote counting to map the direction of effects, alongside narrative thematic synthesis, was used to summarise the findings from the included studies.

**Results:**

Four studies (n = 1,020,342) were included in the review. Three of these studies suggest a possible association between postnatal maternal MHrH and adverse child health outcomes (growth failure, respiratory and gastrointestinal infection) or maltreatment (sexual, physical, and emotional abuse). One study did not find a statistically significant association between postnatal maternal MHrH and infant growth.

**Conclusion:**

The available evidence suggests a possible association between postnatal maternal MHrH and adverse child health and maltreatment outcomes. However, given the small number of studies and methodological heterogeneity, these findings should be interpreted cautiously and highlight the need for further high-quality longitudinal research.

**Systematic Review Registration:**

https://www.crd.york.ac.uk/prospero/, identifier CRD42023446155.

## Introduction

The World Health Organisation (WHO) emphasises the need for mental health for overall well-being (1) and for achieving the “Sustainable Development Goals” (SDGs) (2). Maternal mental health conditions comprise a wide range of conditions that affect a person’s mood, thoughts, emotions, and behaviours. “These conditions included depressive disorders, anxiety disorders, bipolar disorders, eating disorders, and substance misuse (3–5)”. They represent a significant public health issue, affecting the lives of millions of women worldwide (6). The unique triggers associated with pregnancy and the postpartum period place women at heightened risk of developing mental health conditions (7–9). “Dysregulated and disturbed emotions during the postnatal period, including anxiety, frustration, sadness, and guilt, increase women’s vulnerability to multiple mental health conditions” (10). Additionally, biological vulnerabilities, such as fluctuations in neuroendocrine function and hypothalamic-pituitary-adrenal stress hormones, decline sharply during the postnatal period, which increases the risk of mental health conditions during the postpartum period (11, 12). Studies have estimated that 13% to 20% of women experience postnatal mental health conditions worldwide (6). Pre-existing mental health conditions, which occur before and during pregnancy, are the most common risk for the emergence of mental health conditions during the postnatal period (13, 14). Hospital admission rates are frequently used as a population-level measure of mental health-related conditions, with postnatal hospitalisations occurring more frequently than antenatal hospitalisations (15). Postnatal hospitalisations are most commonly associated with severe depression, schizophrenia, schizoaffective disorders, psychotic disorders, substance misuse, and bipolar disorders (16–20).

Postnatal hospitalisation may have far-reaching consequences beyond the mother. Children of affected mothers may be at risk of both short and long-term consequences that affect their development, education, and social well-being (21). These children are more likely to experience adverse health outcomes, such as growth failure, weakened immune function, and increased susceptibility to infections, including lower respiratory infections and gastroenteritis (diarrhoea) (22–24). Furthermore, these children face a higher risk of maltreatment (25–27). Although child outcomes such as child health and maltreatment are often measured separately, they may co-occur. This co-occurrence can be understood within a biopsychosocial framework, which explains the association between postnatal maternal mental health conditions and adverse child outcomes. Within this framework, maternal mental health conditions influence child outcomes through interconnected biological, behavioural, and social pathways (28). Biologically, maternal mental health conditions may influence child outcomes through neuroendocrine dysregulation and altered maternal physiology (29). These processes can contribute to poor feeding practices and compromised immunity. Behaviourally, maternal mental health may disrupt caregiving capacity and mother-child attachment, adversely affecting both child health and safety (21). Socially, maternal mental conditions often co-occur with broader contexts of socio-economic disadvantage, substance misuse, and limited support systems, which can increase the risk of both poor physical health and adverse social outcomes (30). Importantly, child growth failure and susceptibility to infection may also be indicative of neglect, highlighting the overlap between child health outcomes and child maltreatment (31).

Despite the high burden of postnatal maternal MHrH, there are inconsistencies in findings. Some studies have suggested that no direct association exists between postnatal maternal MHrH and adverse child outcomes (32). Additionally, searches of Cochrane, Epistemonikos, and the International Prospective Register of Systematic Reviews (PROSPERO) reveal that no systematic reviews or meta-analyses have been conducted to synthesise the available evidence and address inconsistencies in findings on the association between postnatal maternal MHrH and adverse child health outcomes and child maltreatment. To address this gap and inform opportunities for prevention and intervention, we designed a systematic review to answer the following research question: Are children (Population (P)) whose mothers have one or more MHrH within the 18-month postnatal period (Intervention (I)) at increased risk of adverse child health outcomes or child maltreatment (Outcomes (O)) compared with children whose mothers do not have a MHrH within the 18-month postnatal period (Comparator (C))?

## Methodology

### Protocol registration and reporting

During the protocol formulation, consultation with experts in methodology and systematic review took place. The protocol was subsequently registered on 09 October 2023 with the PROSPERO registration number [CRD42023446155]. The protocol covers two related systematic reviews and meta-analyses conducted by the same author team: the first examines postnatal maternal mental health-related hospitalisation (MHrH) and child developmental and educational outcomes and is currently in press, while the second is the present review, which is under review. While both reviews used similar search strategies and exposure definitions, they addressed distinct research questions and outcomes and were therefore conducted and reported separately to maintain clarity and methodological coherence. The systematic review was reported according to the Preferred Reporting Items for Systematic Review and Meta-Analysis (PRISMA) 2020 checklist (33) (see **Supplementary File (Table 1)**).

### Data source and search strategy

Following consultation with librarians, specific search strategies were designated for each academic database (MEDLINE, PsycINFO, Embase, CINAHL, and Scopus) and Google Scholar from June to August 2023. Utilising the Population, Intervention, Comparison, and Outcome (PICO) format, Medical Subject Headings (MeSH) terms, wildcards, and keywords, we developed search strategies for postnatal maternal MHrH, adverse child health, and child maltreatment. Boolean operators such as “AND” and “OR” were used to combine search terms. A final update search was performed on December 26, 2026, to capture any studies published after our initial search. Additionally, a manual search examined cross-references from eligible papers to uncover potentially relevant articles. A grey literature search was conducted using Google. A detailed summary of the Medline search strategy is provided in the **Supplementary File (Table 2)**.

### Eligibility criteria and outcomes of interest

Observational studies that assessed adverse health or maltreatment outcomes among children whose mothers experienced MHrH within 0–18 months postnatal period based on the Diagnostic and Statistical Manual of Mental Disorders (DSM) or International Classification of Diseases (ICD) diagnostic classifications, were included in the review. While the obstetric definition limits the postpartum period to six weeks after delivery (34), we have followed the convention in maternal mental health research, which defines the postpartum period as up to eighteen months to capture longer-term maternal mental health trajectories and child outcomes (35, 36). In this review, we restricted the exposure to inpatient hospital admission for the following reasons. First, theoretically, severe maternal mental illness has greater potential to disrupt maternal caregiving capacity, attachment, and early child development. So, targeting the highest-risk end of the mental health spectrum, aligning with developmental and psychosocial theories of early adversity (21, 36). Second, this approach enhances diagnostic validity, as inpatient admissions typically involve comprehensive and systematic assessments and recordings in administrative and clinical datasets, providing high reliability, minimal recall bias, and precise timing, which enables accurate temporal linkage with child outcomes. Primary studies were included if they measured adverse child health outcomes using the World Health Organization (WHO) child growth standard (37), clinical diagnosis codes (38, 39), adverse child health questionnaires (40); or if they measured child maltreatment using child abuse potential inventory (41), reliable national datasets records (42), childhood trauma questionnaire (43), adverse childhood experience questionnaire (44), juvenile victimization questionnaire (45), and the parent-child conflict tactics scale (46). This review encompassed studies without restriction on language, geography, or year of publication.

### Study selection

The study selection process involved screening titles and abstracts, followed by full-text screening using a standardised Joanna Briggs Institute (JBI) tool (47). Two authors (DMD and YTK) independently screened the titles and abstracts to identify those meeting the inclusion criteria. One author (BD) examined reference lists to identify potentially relevant articles not included in the initial search strategy. Two authors (BMB and WAB) conducted a full-text review, resolving discrepancies through discussion with a third author (YTK). EndNote version 9 was used to manage the included studies and eliminate duplicates (48).

### Data extraction

An Excel data extraction template using the JBI Data Extraction Tool was created. The template was tested on 50% of the articles and continuously revised as needed. Data extraction started on 30/10/2024. Two authors (DMB and YTK) independently extracted the data from the included studies. The information generated includes study characteristics (first author name, publication year, data collection year, exposure measure, study setting, and study design); participants’ characteristics (child age, sample size, cause of hospitalisation, and period of hospitalisation); and the outcome of interest.

### Methodological quality and risk of bias assessment

Two authors (DMB and BMB) assessed the methodological quality and risk of bias for the included studies. Any discrepancies were resolved through discussion with a third author (WAB). The Newcastle-Ottawa Scale (NOS) quality assessment tool for non-randomised studies was used to evaluate the quality of the included studies (49). Each study was independently rated using the NOS star rating system, with higher scores indicating higher-quality studies. For cross-sectional studies, a rating from 0 to 10 stars is divided into three domains: selection (up to 5 stars), comparability (up to 2 stars), and outcome (up to 3 stars). For cohort studies, ratings range from 0 to 9 stars and are divided into three domains: selection (up to 4 stars), comparability (up to 2 stars), and outcomes (up to 3 stars). Cohort studies were rated as poor (0–2 stars), fair (3–7 stars), or good (8–9 stars), while cross-sectional studies were rated as poor (0–4 stars), fair (5-6), good (7–8 stars), or very good (9–10 stars) (see **Supplementary File (Table 3)**).

### Data synthesis

Given the limited number of included studies and the variability in effect measures (e.g., rate ratio, odds ratio, or risk ratio), exposure measures, outcome measures, follow-up duration, and approaches to confounder adjustment, a meta-analysis was not conducted. Instead, we employed a descriptive vote counting to map the direction of association and thematic data synthesis to synthesise the findings from the included studies (50). This approach involved organising and presenting the data through text, tables, and figures. The findings are summarised across two themes, one for each outcome: the association between postnatal maternal MHrH and adverse child health outcomes, and the association between postnatal maternal MHrH and child maltreatment.

## Results

### Search results

**Figure 1:**
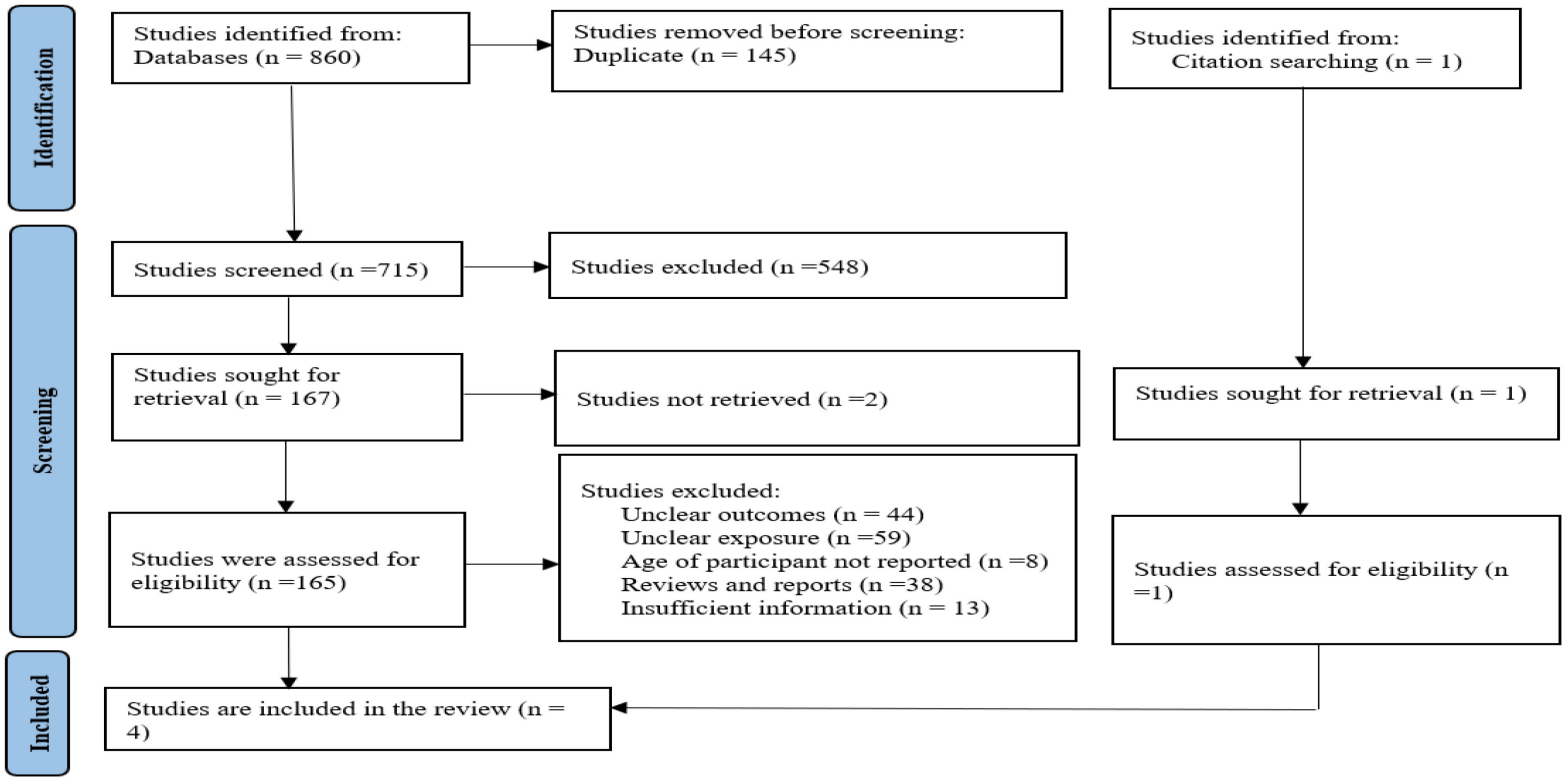
Prisma flow doagram.

Our search generated 861 records, from which duplicates were removed, and screening was conducted, resulting in four eligible studies for this review (**Figure 1**).

### Characteristics of the included studies

This review comprises two cohorts (51, 52) and two cross-sectional studies (32, 53); two studies examined adverse child health outcomes (32, 51), and two studies examined child maltreatment outcomes (52, 53) associated with postnatal maternal MHrH. The studies were conducted across various regions: one in the United States (52), one in Europe (53), one in Australia (51), and one in Africa (32). The sample size of the studies ranged from 147 to 551,232. The included studies used different measures to define postnatal maternal MHrH. One study used the Diagnostic and Statistical Manual of Mental Disorders, Fourth Edition (DSM-IV) (32), and the remaining three studies utilised the International Classification of Diseases (ICD) to report diagnoses of mental health conditions (51–53). For measures of adverse child health outcomes, one study examined age-standardised weight using the Tanner scales (35), and a second study investigated the rate of child admissions to inpatient care due to gastrointestinal and respiratory infections, utilising the International Classification of Diseases (ICD) (51). The remaining two studies focussed on outcomes related to alleged and substantiated reports of child maltreatment from administrative records of child protection services (CPS) (52, 53) (**Table 1**). Moreover, basic descriptive statistics on the composition of the exposed and unexposed groups, together with the proportions of each measured outcome for exposed and unexposed groups, are provided in the **Supplementary File (Table 4)**.

**Table 1:** Characteristics of the included studies.

Author [year]	Study period	Exposure definition	Outcome measure	Sample size	Child age	Effect estimates	Adjusted confounders	Admission period
Tomlinson M, et al., 2006 (32).	2006	Severe postpartum depression	Weight for age	147	2–18 months	Odds ratio	Birth weight	<18 months
Pierce M, et al., 2023 (51).	1980-2001	Admission due to schizophrenia, bipolar, unipolar major depression, paranoid states, or non-organic psychoses	Child admissions due to respiratory or gastrointestinal infection	467,945	5 years	Rate ratio	Parental age, marital status, sex, and birth order of the child, ethnicity, residency, maternal and paternal place of birth, and socio-economic status	<12 months
Hammond I et al., 2017 (52).	2016	Admission due to psychotic, mood, or anxiety disorder.	Alleged maltreatment	551,232	1 year	Risk ratio	Ethnicity, maternal age, parity, prenatal care, insurance type, and paternity establishment	<12 months
Glangeaud F et al., 2013 (53).	2001-2007	Admission due to schizophrenia, other psychotic disorders, bipolar or depressive disorders.	Out of home care placement	1,018	1 year	Odds ratio	Child age, partner’s mental or behavioural disorders	<12 months

### The association between postnatal maternal MHrH and adverse child health outcomes

Two studies of differing quality examined the association between postnatal maternal MHrH and adverse child health outcomes (32, 51). A small, poor-quality cross-sectional study from South Africa (n = 147) reported no statistically significant association between postnatal maternal MHrH and infant growth (32). In contrast, a larger, good quality retrospective cohort study from Western Australia (n = 46,794) found that children whose mothers experienced postnatal MHrH had 22% higher rates of hospitalisation for gastrointestinal infection (adjusted Rate Ratio (aRR) = 1.22; 95% CI; 1.11, 1.33) and a 35% higher rate of admission for respiratory infection (aRR = 1.35; 95% CI; 1.20, 1.51) compared to children whose mothers did not experience postnatal MHrH (51). When the studies are considered together, the evidence appears inconsistent, however with greater weight given to the larger, good-quality study suggests a possible association between postnatal maternal MHrH and adverse child health outcomes.

### The association between postnatal maternal MHrH and child maltreatment

Two studies examined the relationship between postnatal maternal MHrH and child maltreatment. A retrospective cohort study in California, USA, reported that, after controlling for socio-demographic factors, children whose mothers experienced postnatal MHrH had 5.69 times higher risk of maltreatment compared to children whose mothers did not experience postnatal MHrH (adjusted Risk Ratio (aRR) = 5.69; 95% CI: 5.51, 5.87) (52). A study in France and Belgium with 1,018 children reported that mothers with postnatal MHrH had 4.5 times higher odds of child maltreatment than children without hospitalisation (aOR = 4.5; 95% CI: 1.7, 12.4) (53).

### Quality of the included studies

One study was given a good quality rating (51), two studies were considered fair quality (52, 53), and one study was categorised as poor quality (32). The study of good quality had used standardised tools to measure “exposures/outcomes” and adjusted for the most important confounders. This study had a clearly defined and representative cohort, with exposure and outcomes not present at the start of the study. The studies rated as fair quality used a “representative sample” but needed greater rigour when defining or assessing “exposures/outcomes”. The authors adjusted for some but not all important “confounders”. While “outcome assessments” were not fully blinded, efforts were made to ensure objective evaluation. One study categorised as “poor quality” lacked clear “eligibility criteria” and made a light adjustment for “confounders.

## Discussion

This is the first systematic review to synthesise the evidence on the association between postnatal maternal MHrH and the risk of adverse child health and maltreatment outcomes. Although we employed a comprehensive search strategy across multiple databases and grey literature sources, the review yielded only a limited number of eligible studies. This likely reflects a genuine gap in the literature and underscores the need for further primary research. Recent research indicates a growing interest in this field; however, much of it falls outside our inclusion criteria due to differences in the definition of maternal mental health conditions. For example, Subbiah et al. (2025) assessed postnatal maternal mental health condition using self-reported screening tools and found an association with infants’ health-related quality of life (54). Similarly, Hellyer et al. (2025) conducted a systematic review and meta-analysis focussing on non-hospitalised postnatal depression beyond the 12-month postpartum period (55).

The good-quality study included in this review provides evidence of a potential association between postnatal maternal MHrH and adverse child health outcomes (51). This finding implies that the potential impact of postnatal maternal MHrH may extend beyond immediate child health outcomes and have lifelong implications for wellbeing and physical health (56–59). In contrast, a poor-quality study did not find a statistically significant association between postnatal maternal MHrH and infant growth (32), highlighting inconsistency in the available evidence. The included studies in this review also provide evidence of a potential association between postnatal maternal MHrH and child maltreatment (52, 53). One study further indicated that comorbid maternal substance misuse may increase the likelihood of child maltreatment within the postnatal period (52, 53).

Findings from this systematic review reinforce existing evidence-based recommendations for early screening, comprehensive psychosocial assessment, and the provision of adequate, timely, and family-centred follow-up care and support in community settings to prevent and mitigate the impact of maternal mental health issues on child outcomes. Importantly, effective identification and responses to maternal mental health issues in the perinatal period must go beyond treating the individual mother alone. Screening before, during, and after pregnancy, combined with a whole family approach that addresses both parental mental health issues and improves mother-child interaction, is essential (21, 60, 61). However, some women may be reluctant to disclose their parenting or family circumstances due to stigma or fear of child protection involvement (62). Therefore, effective support requires a sensitive, respectful, and holistic approach that acknowledges the complexities of parental mental health conditions and prioritises the needs of mothers, children, and their families (63).

Where hospitalisation is considered necessary, it is important that mothers have access to holistic, multidisciplinary care through specialist mother-baby units, where co-admission of children is supported (64). This ensures that mothers have access to specialist inpatient care and treatment, while remaining with and being supported to continue providing care for their child. Mother-baby units deliver comprehensive, whole-family-centred care aimed at strengthening mother-infant relationships and supporting parenting capacity. Within the unit, ongoing physical, developmental, and socio-emotional assessments of the infant facilitate early identification of health and socio-emotional-related problems, enabling timely interventions (65). Evidence indicates that mother-baby units are associated with improvements in maternal mental health and a range of child outcomes (65).

This review has the following limitations. The current evidence is derived from observational studies that focus solely on the postnatal period, without considering the broader perinatal mental health trajectory. Maternal mental health problems often begin before or during pregnancy and evolve over time, suggesting that postnatal MHrH reflects one point along a continuum of vulnerability rather than a distinct exposure (66–68). Prenatal and antenatal psychological and emotional factors are associated with later child outcomes (69), and social and contextual factors such as socio-economic disadvantage and limited social support often persist across the perinatal period, shaping both maternal mental health and child outcomes (70). Excluding the prenatal and antenatal period, therefore, limits conceptual completeness and makes it difficult to determine whether postnatal MHrH independently affects child outcomes or reflects cumulative maternal mental health burden. Longitudinal designs that capture maternal mental health across the pre-pregnancy, antenatal, and postnatal periods are needed. Heterogeneity arising from the small number of studies, differences in study quality, and methodological variation, limits the interpretability and generalisability of findings. Differences in exposure definitions, diagnostic systems, and timing of exposure reflect variability in the severity and chronicity of MHrH, potentially introducing misclassification and biasing associations with child outcomes (21, 71). Restricting the exposure definition to inpatient psychiatric admission may limit the generalisability of the findings and potentially underestimate the overall population impact, particularly among mothers treated in outpatient or community settings. Heterogeneity also arises from differences in outcome assessment tools and timing of measurement (ranging from 2 months to 5 years). The mechanisms linking maternal mental health to child outcomes are likely to differ across developmental stages (72). Outcomes in early infancy may be more strongly influenced by caregiving capacity and maternal-infant interaction, whereas outcomes in early childhood may reflect cumulative exposure to social and environmental risks (21, 73). Consequently, combining findings across these periods may mask time-specific effects and reduce the interpretability. These sources of heterogeneity have important implications for inference, limiting the ability to draw robust conclusions about the strength, direction, and consistency of the associations between postnatal MHrH and child outcomes (74). Given this variability, a meta-analysis was not undertaken (75). Instead, a structured narrative synthesis, supported by a synthesis matrix (76), was used to identify patterns across studies, which were generally consistent in direction (see **Supplementary File (Table 5)**). Moreover, the protocol was not strictly prospective, and minor methodological refinements were made. Web of Science was removed because most of the articles it indexes are also covered by other databases, and Google Scholar was included to capture potentially relevant grey literature. Date and language restrictions were removed to minimise selection and publication bias. Importantly, these refinements are unlikely to have affected the study selection, evidence synthesis, or overall conclusion of the review.

## Conclusion

The available evidence suggests a possible association between postnatal maternal MHrH and adverse child health and maltreatment outcomes. However, given the small number of studies and methodological heterogeneity, these findings should be interpreted cautiously and highlight the need for further high-quality longitudinal research.

## Data Availability

The original contributions presented in the study are included in the article/**Supplementary Material**. Further inquiries can be directed to the corresponding author.
