# Supplementary material for: Postnatal maternal mental health-related hospitalisation and its association with adverse child health and maltreatment outcomes: narrative review
Source: Front Psychiatry. 2026 Jun 8;17:1850017. doi: 10.3389/fpsyt.2026.1850017 (PMC13283991; doi:10.3389/fpsyt.2026.1850017)
Supplement: Supplementary file 1 [file SupplementaryFile1.docx]

**Supplementary file**

Table 1: PRISMA checklist

Table 2: Search strategies for the MEDLINE database

Table 3: Newcastle Ottawa Scale quality assessment tool

Table 4: Descriptive statistics of the exposed and unexposed groups, including the proportions of measured outcomes for exposed and unexposed groups across the included studies

Table 5: Synthesis matrix indicating where exposure outcome combination yields consistent versus divergent results

**Table 1: PRISMA checklist**

| Section | Item # | Checklist item | Location |
| --- | --- | --- | --- |
| TITLE | | |  |
| Title | 1 | Identify the report as a systematic review. | Title |
| ABSTRACT | | |  |
| Abstract | 2 | See the PRISMA 2020 for Abstracts checklist. | Abstract |
| INTRODUCTION | | |  |
| Rationale | 3 | Describe the rationale for the review in the context of existing knowledge. | Introduction |
| Objectives | 4 | Provide an explicit statement of the objective(s) or question(s) the review addresses. |  |
| METHODS | | |  |
| Eligibility criteria | 5 | Specify the inclusion and exclusion criteria for the review and how studies were grouped for the syntheses. | Eligibility criteria and outcomes of interest |
| Information sources | 6 | Specify all databases, registers, websites, organisations, reference lists and other sources searched or consulted to identify studies. Specify the date when each source was last searched or consulted. | Data source and search Strategy |
| Search strategy | 7 | Present the full search strategies for all databases, registers and websites, including any filters and limits used. |  |
| Selection process | 8 | Specify the methods used to decide whether a study met the inclusion criteria of the review, including how many reviewers screened each record and each report retrieved, whether they worked independently, and if applicable, details of automation tools used in the process. | Study selection |
| Data collection process | 9 | Specify the methods used to collect data from reports, including how many reviewers collected data from each report, whether they worked independently, any processes for obtaining or confirming data from study investigators, and if applicable, details of automation tools used in the process. | Data extraction |
| Data items | 10a | List and define all outcomes for which data were sought. Specify whether all results that were compatible with each outcome domain in each study were sought (e.g. for all measures, time points, analyses), and if not, the methods used to decide which results to collect. | Eligibility criteria and outcomes of interest |
|  | 10b | List and define all other variables for which data were sought (e.g. participant and intervention characteristics, funding sources). Describe any assumptions made about any missing or unclear information. |  |
| Study risk of bias assessment | 11 | Specify the methods used to assess risk of bias in the included studies, including details of the tool(s) used, how many reviewers assessed each study and whether they worked independently, and if applicable, details of automation tools used in the process. | Methodological quality and risk of bias assessment |
| Effect measures | 12 | Specify for each outcome the effect measure(s) (e.g. risk ratio, mean difference) used in the synthesis or presentation of results. | Data synthesis |
| Synthesis methods | 13a | Describe the processes used to decide which studies were eligible for each synthesis (e.g. tabulating the study intervention characteristics and comparing against the planned groups for each synthesis (item #5)). | Data synthesis |
|  | 13b | Describe any methods required to prepare the data for presentation or synthesis, such as handling of missing summary statistics, or data conversions. |  |
|  | 13c | Describe any methods used to tabulate or visually display results of individual studies and syntheses. |  |
|  | 13d | Describe any methods used to synthesize results and provide a rationale for the choice(s). If meta-analysis was performed, describe the model(s), method(s) to identify the presence and extent of statistical heterogeneity, and software package(s) used. |  |
|  | 13e | Describe any methods used to explore possible causes of heterogeneity among study results (e.g. subgroup analysis, meta-regression). |  |
|  | 13f | Describe any sensitivity analyses conducted to assess robustness of the synthesized results. |  |
| Reporting bias assessment | 14 | Describe any methods used to assess risk of bias due to missing results in a synthesis (arising from reporting biases). | N/A |
| Certainty assessment | 15 | Describe any methods used to assess certainty (or confidence) in the body of evidence for an outcome. | N/A |
| RESULTS | | |  |
| Study selection | 16a | Describe the results of the search and selection process, from the number of records identified in the search to the number of studies included in the review, ideally using a flow diagram. | Search results |
|  | 16b | Cite studies that might appear to meet the inclusion criteria, but which were excluded, and explain why they were excluded. |  |
| Study characteristics | 17 | Cite each included study and present its characteristics. | Characteristics of the included studies |
| Risk of bias in studies | 18 | Present assessments of risk of bias for each included study. | Quality of the included studies |
| Results of individual studies | 19 | For all outcomes, present, for each study: (a) summary statistics for each group (where appropriate) and (b) an effect estimates and its precision (e.g., confidence/credible interval), ideally using structured tables or plots. | Table 1 |
| Results of syntheses | 20a | For each synthesis, briefly summarise the characteristics and risk of bias among contributing studies. | The association between postnatal maternal MHrH and adverse child health outcomes, and the association between postnatal maternal MHrH and child maltreatment |
|  | 20b | Present the results of all statistical syntheses conducted. If a meta-analysis was done, present, for each, the summary estimate and its precision (e.g., confidence/credible interval), and measures of statistical heterogeneity. If comparing groups, describe the direction of the effect. |  |
|  | 20c | Present the results of all investigations of possible causes of heterogeneity among study results. |  |
|  | 20d | Present the results of all sensitivity analyses conducted to assess the robustness of the synthesized results. |  |
| Reporting biases | 21 | Present assessments of risk of bias due to missing results (arising from reporting biases) for each synthesis assessed. | N/A |
| Certainty of evidence | 22 | Present assessments of certainty (or confidence) in the body of evidence for each outcome assessed. | N/A |
| DISCUSSION | | |  |
| Discussion | 23a | Provide a general interpretation of the results in the context of other evidence. | Discussion |
|  | 23b | Discuss any limitations of the evidence included in the review. |  |
|  | 23c | Discuss any limitations of the review processes used. |  |
|  | 23d | Discuss implications of the results for practice, policy, and future research. |  |
| OTHER INFORMATION | | |  |
| Registration and protocol | 24a | Provide registration information for the review, including register name and registration number, or state that the review was not registered. | Protocol registration and reporting |
|  | 24b | Indicate where the review protocol can be accessed, or state that a protocol was not prepared. |  |
|  | 24c | Describe and explain any amendments to information provided at registration or in the protocol. |  |
| Support | 25 | Describe sources of financial or non-financial support for the review, and the role of the funders or sponsors in the review. | Declarations |
| Competing interests | 26 | Declare any competing interests of review authors. |  |
| Availability of data, code, and other materials | 27 | Report which of the following are publicly available and where they can be found: template data collection forms; data extracted from included studies; data used for all analyses; analytic code; any other materials used in the review. |  |

**Table 2: Search strategies for the MEDLINE database**

| **Adverse child health outcomes** | | | |
| --- | --- | --- | --- |
| # | | Query | Results |
| S7 | | S1 AND S2 AND S3 AND S4 AND S5 AND S6 | 256 |
| S6 | | AB ( breastfeeding OR Diarrhea OR Respiratory infection OR *nutrition OR Acute febrile illness OR Malaria OR Measles OR Pneumonia OR Illness OR *weight OR Obesity OR Stunted OR Wasted OR Growth impairment OR Health status OR Health outcomes ) OR TI ( breastfeeding OR Diarrhea OR Respiratory infection OR *nutrition OR Acute febrile illness OR Malaria OR Measles OR Pneumonia OR Illness OR *weight OR Obesity OR Stunted OR Wasted OR Growth impairment OR Health status OR Health outcomes ) OR SU ( breastfeeding OR Diarrhea OR Respiratory infection OR *nutrition OR Acute febrile illness OR Malaria OR Measles OR Pneumonia OR Illness OR *weight OR Obesity OR Stunted OR Wasted OR Growth impairment OR Health status OR Health outcomes ) | 3,724,008 |
| S5 | | AB ( Child* OR Infant OR Toddler OR Newborn OR Neonates ) OR TI ( Child* OR Infant OR Toddler OR Newborn OR Neonates) OR SU ( Child* OR Infant OR Toddler OR Newborn OR Neonates) | 3,885,526 |
| S4 | | AB ( post* OR puerperium OR *birth OR pregnan* OR parturient OR *delivery OR *gestation OR *conception ) OR SU ( post* OR puerperium OR *birth OR pregnan* OR parturient OR *delivery OR *gestation OR *conception ) OR TI ( post* OR puerperium OR *birth OR pregnan* OR parturient OR *delivery OR *gestation OR *conception ) | 5,930,627 |
| S3 | | AB ( admission OR hospitali*OR Inpatient OR readmission OR “Psychiatric contact” OR “mental health service use”) OR TI ( admission OR hospitali*OR Inpatient OR readmission OR “Psychiatric contact” OR “mental health service use” ) OR SU ( admission OR hospitali*OR Inpatient OR readmission OR “Psychiatric contact” OR “mental health service use” ) | 406,079 |
| S2 | | AB (mental W1 disorder OR “mental illness” OR adjustment disorder OR affective disorder OR dysthymic disorder OR psychiatr* disorder OR behavio* disorder OR psychological disorder OR depress* N1 disorder OR stress W1 disorder OR anxiety N2 disorder OR mood disorder OR PTSD OR post-traumatic stress disorder OR psychos* N1 disorder OR *polar disorder OR schizo* W1 disorder OR emotional disorder OR neuro* disorder OR nervous disorder ) OR TI ( mental W1 disorder OR “mental illness” OR adjustment disorder OR affective disorder OR dysthymic disorder OR psychiatr* disorder OR behavio* disorder OR psychological disorder OR depress* N1 disorder OR stress W1 disorder OR anxiety N2 disorder OR mood disorder OR PTSD OR post-traumatic stress disorder OR psychos* N1 disorder OR *polar disorder OR schizo* W1 disorder OR emotional disorder OR neuro* disorder OR nervous disorder ) OR SU ( mental W1 disorder OR “mental illness” OR adjustment disorder OR affective disorder OR dysthymic disorder OR psychiatr* disorder OR behavio* disorder OR psychological disorder OR depress* N1 disorder OR stress W1 disorder OR anxiety N2 disorder OR mood disorder OR PTSD OR post-traumatic stress disorder OR psychos* N1 disorder OR *polar disorder OR schizo* W1 disorder OR emotional disorder OR neuro* disorder OR nervous disorder ) | 378,372 |
| S1 | | AB ( mother OR matern* OR wom? n ) OR SU ( mother OR matern* OR wom?n ) OR TI ( mother OR matern* OR wom? n ) | 2,264,066 |
| **Child maltreatment** | | | |
| # | Query | | Results |
| S7 | (S1 AND S2 AND S3 AND S4 AND S5 AND S6) | | 64 |
| S6 | AB ( Foster care OR out-of-home care OR OHC OR allegations OR substantiations OR care replacement OR protection OR detention OR maltreatment OR notification OR neglect OR abuse ) OR SU ( Foster care OR out-of-home care OR OHC OR allegations OR substantiations OR care replacement OR protection OR detention OR maltreatment OR notification OR neglect OR abuse ) OR TI ( Foster care OR out-of-home care OR OHC OR allegations OR substantiations OR care replacement OR protection OR detention OR maltreatment OR notification OR neglect OR abuse ) | | 758,384 |
| S5 | AB ( Child* OR Infant OR Toddler OR Newborn OR Neonates ) OR TI ( Child* OR Infant OR Toddler OR Newborn OR Neonates) OR SU ( Child* OR Infant OR Toddler OR Newborn OR Neonates) | | 3,885,862 |
| S4 | AB ( post* OR puerperium OR *birth OR pregnan* OR parturient OR *delivery OR *gestation OR *conception ) OR SU ( post* OR puerperium OR *birth OR pregnan* OR parturient OR *delivery OR *gestation OR *conception ) OR TI ( post* OR puerperium OR *birth OR pregnan* OR parturient OR *delivery OR *gestation OR *conception ) | | 5,931,575 |
| S3 | AB ( admission OR hospitali*OR Inpatient OR readmission OR “Psychiatric contact” OR “mental health service use”) OR TI ( admission OR hospitali*OR Inpatient OR readmission OR “Psychiatric contact” OR “mental health service use” ) OR SU ( admission OR hospitali*OR Inpatient OR readmission OR “Psychiatric contact” OR “mental health service use” ) | | 406,079 |
| S2 | AB (mental W1 disorder OR “mental illness” OR adjustment disorder OR affective disorder OR dysthymic disorder OR psychiatr* disorder OR behavio* disorder OR psychological disorder OR depress* N1 disorder OR stress W1 disorder OR anxiety N2 disorder OR mood disorder OR PTSD OR post-traumatic stress disorder OR psychos* N1 disorder OR *polar disorder OR schizo* W1 disorder OR emotional disorder OR neuro* disorder OR nervous disorder ) OR TI ( mental W1 disorder OR “mental illness” OR adjustment disorder OR affective disorder OR dysthymic disorder OR psychiatr* disorder OR behavio* disorder OR psychological disorder OR depress* N1 disorder OR stress W1 disorder OR anxiety N2 disorder OR mood disorder OR PTSD OR post-traumatic stress disorder OR psychos* N1 disorder OR *polar disorder OR schizo* W1 disorder OR emotional disorder OR neuro* disorder OR nervous disorder ) OR SU ( mental W1 disorder OR “mental illness” OR adjustment disorder OR affective disorder OR dysthymic disorder OR psychiatr* disorder OR behavio* disorder OR psychological disorder OR depress* N1 disorder OR stress W1 disorder OR anxiety N2 disorder OR mood disorder OR PTSD OR post-traumatic stress disorder OR psychos* N1 disorder OR *polar disorder OR schizo* W1 disorder OR emotional disorder OR neuro* disorder OR nervous disorder ) | | 913,052 |
| S1 | AB ( mother OR matern* OR wom?n ) OR SU ( mother OR matern* OR wom?n ) OR TI ( mother OR matern* OR wom?n ) | | 2,264,066 |

**Table 3: Newcastle Ottawa Scale quality assessment tool**

| Authors (year) | Representativeness of the exposed cohort | Selection of the non-exposed cohort | Ascertainment of the exposure | The outcome was not present at the start of the study | Comparability of cohorts | Assessment of outcome | Follow up long enough for an outcome to occur | Adequacy of follow-up of the cohort | Quality |
| --- | --- | --- | --- | --- | --- | --- | --- | --- | --- |
| Pierce M, et al., 2023 (51) | * | * | * | * | * | * | * | * | Good |
| Hammond I, et al., 2017 (52) | * | * | * | * | * | * | * | - | Fair |
| Glangeaud NM, et al., 2013 (53) | * | - | * | * | * | * | - | * | Fair |
| Cross-sectional studies | | | | | | | | | |
|  | Representativeness of the sample | Sample size | Non-respondents | Ascertainment of the exposure | Confounder controlled | Assessment of outcome | Statistical test |  |  |
| Tomlinson M, et al., 2006 (32) | - | - | - | ** | - | ** | - |  | Poor |

**Table 4: Descriptive statistics of the exposed and unexposed groups, including the proportions of measured outcomes for exposed and unexposed groups across the included studies**

| Author [year] | Details of the included studies are provided |
| --- | --- |
| Tomlinson M, et al., 2006 (32). | -Depression was found in 34.7% of individuals two months after childbirth and in 12% of individuals eighteen months postpartum. |
| Pierce M, et al., 2023 (51). | -Approximately 2% of the children belonged to the exposed group. -Children in this group experienced a 70% higher relative rate of hospital admissions compared to those who were not exposed.  -Within the exposed group, 2.37% were hospitalised due to respiratory infections, while 2.51% were admitted for gastrointestinal infections. |
| Hammond I et al., 2017 (52). | -Roughly 3% of the children belonged to the exposed group.  -Within this group, 41.3% had mothers with recorded instances of maternal substance abuse.  -Approximately 36% of the exposed children were involved with CPS.  -Conversely, only 4.4% of the non-exposed children had any CPS involvement. |
| Glangeaud F. et al., 2013 (53). | -Around 15% of exposed children had out-of-home care placement |

**Table 5: Synthesis matrix indicating where exposure outcome combination yields consistent results**

| **Study** | **Direction of Association** | **Consistency** |
| --- | --- | --- |
| Tomlinson M, et al., 2006 | No association between postnatal MHrH and adverse child health, such as child growth. | Null |
| Pierce M, et al., 2023 | Positive association between postnatal MHrH and child admission for infection. | Consistent |
| Hammond et al., 2017 | Positive association between postnatal MHrH and increased risk of child maltreatment. | Consistent |
| Glangeaud NM, et al., 2013 | Positive association between postnatal MHrH and increased risk of child maltreatment. | Consistent |
